# Targeting Glutamine Metabolism as an Attractive Therapeutic Strategy for Acute Myeloid Leukemia

**DOI:** 10.1007/s11864-023-01104-0

**Published:** 2023-05-30

**Authors:** Yan Xiao, Bingbing Hu, Yao Guo, Dengyang Zhang, Yuming Zhao, Yun Chen, Na Li, Liuting Yu

**Affiliations:** 1 Edmond H. Fischer Translational Medical Research Laboratory, Scientific Research Center, the Seventh Affiliated Hospital, Sun Yat-Sen University, Shenzhen, China; 2 Reproductive Medicine Center, the Seventh Affiliated Hospital, Sun Yat-Sen University, Shenzhen, China; 3 Department of Nephrology, Center of Kidney and Urology, the Seventh Affiliated Hospital, Sun Yat-Sen University, Shenzhen, China

**Keywords:** Acute myeloid leukemia, Cancer metabolism, Glutamine, Glutaminase inhibitor, Glutamine antagonist, AML treatment

## Abstract

Relapse after chemotherapy and hematopoietic stem cell transplantation leads to adverse prognosis for acute myeloid leukemia (AML) patients. As a “conditionally essential amino acid,” glutamine contributes to the growth and proliferation of AML cells. Glutamine-target strategies as new treatment approaches have been widely explored in AML treatment to improve outcome. Glutamine-target strategies including depletion of systemic glutamine and application of glutamine uptake inhibitors, glutamine antagonists/analogues, and glutaminase inhibitors. Because glutamine metabolism involved in multiple pathways in cells and each pathway of glutamine metabolism has many regulatory factors, therefore, AML therapy targeting glutamine metabolism should focus on how to inhibit multiple metabolic pathways without affecting normal cells and host immune to achieve effective treatment for AML.

## Introduction

Acute myeloid leukemia (AML) is a type of heterogeneous hematologic malignancy, which is characterized by impaired clonal proliferation and differentiation of hematopoietic cells in bone marrow [1, 2]. In the past decades, the “7 + 3” induction chemotherapy (cytarabine for 7 days + daunorubicin for 3 days) has been usual option for AML treatment. It turns out that the AML patients at young age benefit greatly from this therapy. Although patients with AML can achieve remission with intensive chemotherapy, the drug-resistant relapse leads to an unsatisfactory overall 5-year survival rate (20–30%) [3, 4]. The clinical application of allogeneic hematopoietic stem cell transplantation contributes to improved long-term survival of AML patients, but post-transplant relapse could occur due to multiple factors. Therefore, more alternative therapeutic strategies for AML patients are needed.

Malignant cells grow and divide faster and more efficiently than normal cells, which increase their demand for energy, biosynthetic precursors, and macromolecular synthesis [5]. Energy metabolic reprogramming promotes the proliferation of cancer cells. Studies have showed that cancer cells preferentially use glycolysis to rapidly generate ATP for cell growth and proliferation even under the condition with sufficient oxygen, which is known as Warburg effect. In addition to glycolysis, various metabolic pathways including glutamine decomposition are activated in cancer cells [6, 7]. Glutamine metabolism plays a key role in cancer cells, providing the basis for regulating redox homeostasis and signal transduction pathways, and maintaining cell proliferation [8].

This review highlights the importance of glutamine metabolism in AML cells and addresses potential related strategies for AML treatment, including depletion of systemic glutamine and application of glutamine uptake inhibitors, glutamine antagonists/analogues, and glutaminase inhibitors. The opportunities and challenges in targeting glutamine metabolism are also discussed.

## Importance of glutamine in AML cells

Glutamine is the most abundant non-essential amino acid in human blood with a concentration at 0.6 to 0.8 mM [8]. Normally, glutamine can be transported from the outside of cells through glutamine transporter SLC1A5 (ASCT2), synthesized within cells or produced by lysosomal degradation of proteins obtained by autophagy, endocytosis, and macrocytosis. However, glutamine is considered as a conditionally essential amino acid when de novo synthesis of glutamine cannot meet the energy demand for the rapid proliferation of cancer cells. The central role of glutamine in metabolism stems from the various metabolic pathways converting glutamine to α-KG [8, 9]. Firstly, glutamine can be converted to glutamate by glutaminase (GLS). On one hand, glutamate can be directly converted to α-KG by glutamate dehydrogenase (GLDH), which was involved in the tricarboxylic acid (TCA) cycle as a metabolite for cancer cell growth and proliferation. On the other hand, glutamate can be deaminated in many reactions and thus used as a nitrogen source for the non-essential amino acids and purine and pyrimidine nucleotides [10]. In addition, intracellular glutathione derived from glutamine is a vital small molecule reductant that can effectively scavenge intracellular reactive oxygen species (ROS) and contribute to the redox homeostasis of cancer cells [11]. Moreover, glutamine can promote activation of rapamycin complex 1 (mTORC1), which is associated with apoptosis and autophagy of cancer cells [12, 13].

The plasma glutamine concentration of AML patients is significantly lower than 0.3 mM, revealing the rapid consumption of glutamine in AML cells [14]. Wang D et al. analyzed the serum metabolites of 55 newly diagnosed AML patients and 45 matched healthy volunteers. The results indicated that glutamine was decreased in AML patients in comparison to that in healthy subjects [15]. Importantly, glutamine acted as the main energy source for the growth and proliferation of AML cells. Evi1/MF9AML cells can use glutamine metabolites to accelerate cell oxidative phosphorylation prior to glycolysis activation, indicating that AML cells are more dependent on glutamine [16]. Therefore, inhibition of glutamine metabolism is suggested as a therapeutic method to prevent the growth of leukemia cells. Actually, glutamine deprivation suppressed the proliferation and growth of AML cells significantly [17–21]. Emadi’s group also uncovered that reducing glutamine resulted in a decrease of the metabolite 2-hydroxybutyric acid (2-HG) and thus inhibited the proliferation of AML cells with isocitrate dehydrogenase (IDH) mutations [18].

Recently, several potential anti-AML targets related to glutamine metabolism in AML cells have been predicted and confirmed. Maria LA and colleagues found that STAT3 mediated oxidative phosphorylation in leukemia stem cells (LSCs) by regulating MYC expression and thus modulate the transcription of SLC1A5 that was involved in glutamine metabolism and TCA cycle [22]. Additionally, insulin-like growth factor 2 mRNA-binding protein 2 (IGF2BP2) facilitated AML development and LSCs self-renewal through regulating the expression of key targets in the glutamine metabolic pathway [23]. Zhao H et al. revealed that the anti-leukemia drug Chidamide, an oral active blocker of histone deacetylase (HDAC), reduced the uptake of glutamine and exerted cytotoxicity on LSC-like cells [24]. Taken together, these studies indicate that targeting glutamine metabolism can play an anti-leukemia role in AML cells. Currently, there are many strategies for targeting glutamine metabolism in cancer therapy, such as directly depriving glutamine, blocking glutamine transporters, or inhibiting glutaminase activity in cancer cells.

## Targeting glutamine metabolism in AML cells

**Fig. 1:**
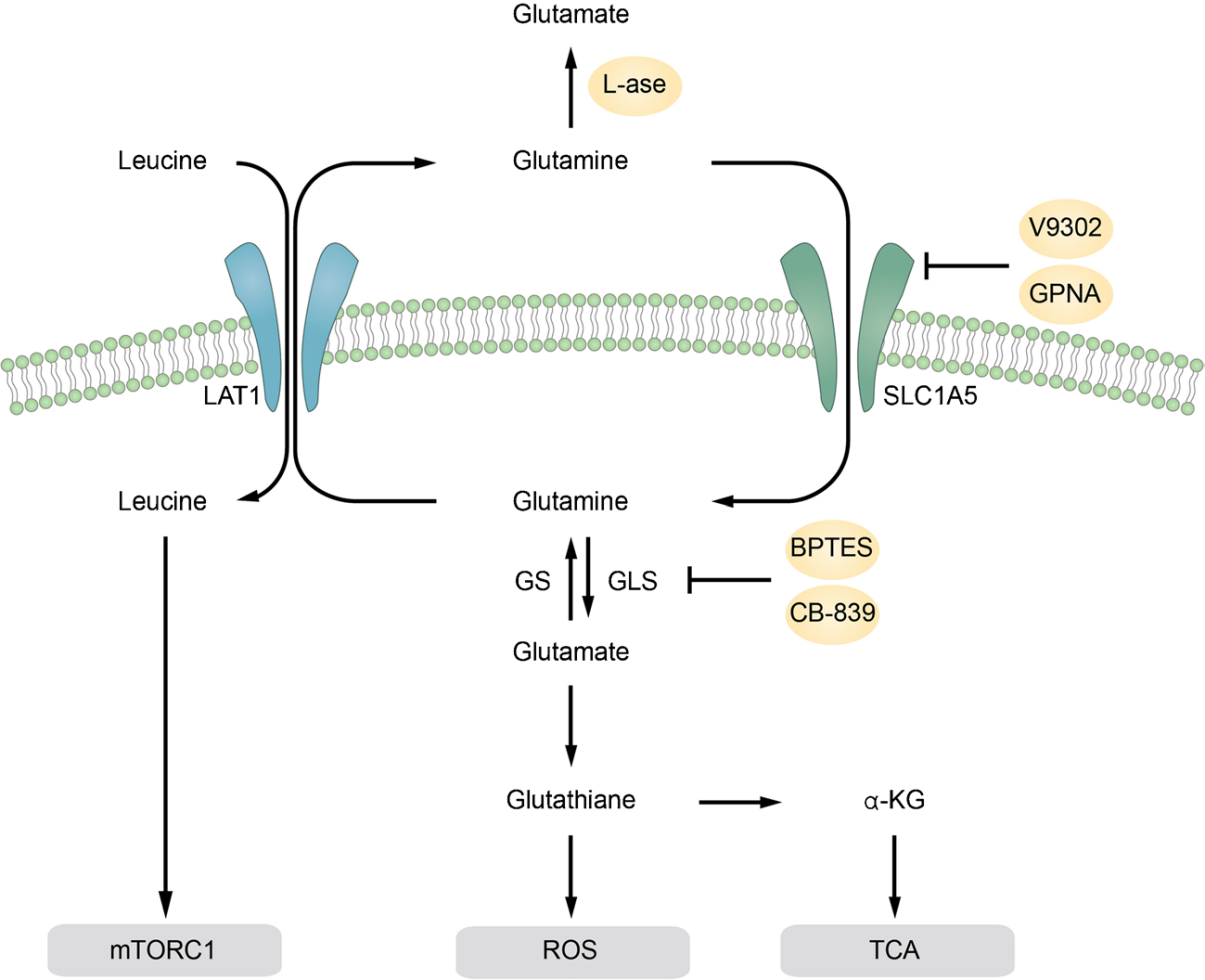
Strategies of targeting glutamine metabolism in AML. Application of L-asparaginase hydrolyzes extracellular glutamine while glutamine transporter SLC1A5 inhibitors including V9302 and GPNA can block the uptake of glutamine into cells. Consequently, the intracellular glutamine is decreased, leading to the reduction of leucine uptake and inhibition of mTORC1. Glutaminase inhibitors including CB-839 and BPTES can inhibit the activity of glutaminase (GLS) and thus prevent the production of glutamate and glutathione, which results in the ROS accumulation and the inhibition of TCA cycle pathway.

In view of the close relationship between glutamine metabolism and AML development and progression, we have described several strategies for targeting glutamine metabolism in AML cells (Fig. 1), and summarized the related developing drugs for AML (Table 1).

**Table 1:** Drugs targeting on glutamine metabolism in AML

Classification	Drugs	Status
	Erwinaze	Phase I clinical trial [25]
Asparaginase	Pegcrisantaspase	Phase I clinical trial [26]
	ASNase	Phase II clinical trial [27]
Glutamine uptake inhibitors	V-9302	Preclinical tool [28, 29]
	GPNA	Limited by toxicity [30]
Glutamine antagonists/analogues	DON	Limited by toxicity [31–33]
	Acivicin	Limited by toxicity [31, 34]
Glutaminase inhibitors	BPTES	Preclinical tool [5, 18, 35]
	CB-839	Phase I/II clinical trial [36–38]

### Systemic glutamine depletion

One of the most important strategies to treat AML is to reduce the glutamine content in AML cells. Previous studies have identified that leukemic cells make use of glutamine to produce α-KG required for the TCA cycle [10, 12]. Moreover, decreased glutamine levels inhibited mTORC1 activation and promoted apoptosis in leukemic cells [39, 40]. Emadi and colleagues investigated the effects of glutamine depletion on cell growth in a group of acute leukemia cells. It turned out that glutamine deprivation suppressed cell proliferation and induced apoptosis in all cell lines tested [18]. L-asparaginase (L-asp), an enzyme capable of hydrolyzing L-asparagine and L-glutamine, is considered as an effective drug for treating patients with ALL. It exhibits strong anticancer activity due to its ability of inducing autophagy in cancer cells [41]. In recent years, more and more studies have proved that L-asp can exert a good anti-AML effect. Willems et al. uncovered that L-asp reduced glutamine and thus inhibited mTORC1 activity in AML [39]. Furthermore, L-asp was able to facilitate apoptosis through inducing autophagy in AML cell lines such as U937, HL-60, and KG-1a [42]. In addition, L-asp can restrain cellular oxidative phosphorylation and thus inhibit the proliferation of AML cells and improve the prognosis of refractory leukemia [16]. These results suggest that L-asp-induced glutamine depletion may play an important role in the treatment of AML patients.

A number of clinical trials are currently underway to evaluate the effects of L-asp in AML treatment. At present, the L-asp available in clinic mainly comes from two kinds of bacteria, namely *Escherichia coli* and *Erwinia Chrysanthemi* both of which have been proved to have high glutaminase activity. Erwinaze is a clinically available L-asp agent with strong glutaminase activity. Several studies have confirmed the ability of Erwinaze to reduce plasma glutamine levels and improve overall survival in patients with refractory AML [25]. Another long-acting L-asp agent, PegCrisantaspase (PegC), is currently undergoing preclinical and clinical studies in AML and ALL, respectively. PegC was shown to inhibit the phosphorylation of p70S6K and 4EBP1 by decreasing plasma glutamine. It has synergistic effects with BCL-2 inhibitor venetoclax in the treatment of leukemia [26]. ASNase, the first-line chemotherapeutic agent for ALL, functions well as an anti-AML agent [27]. It plays a key role in the chemotherapy for young patients with leukemia. However, the efficacy of ASNase is greatly reduced due to its cytotoxicity in elderly patients. Recently, a new L-asp GRASPA wrapped in red blood cells provides better safety and seems to have better efficacy for elderly patients with leukemia. Currently, the drug has been in phase II clinical trial (ClinicalTrials.gov:NCT01810705).

Overall, L-asp may offer promising new therapeutic strategies for AML treatment. Additionally, L-asp can be combined with methotrexate for the treatment of pediatric refractory/relapsed AML or with higher doses of cytarabine for AML therapy [43, 44]. Although L-asp is effective in individual AML patients, L-asp as a single agent has so far failed to induce complete remission of AML. Holland et al. found that L-asp treatment in AML patients resulted in varying levels of complications and relapse after 8 months, even though patients initially achieved complete remission [45]. Another study from Holland’s group found that L-asp treatment did not reduce the uptake of cysteine, glutamine, histidine, or tyrosine in AML cells [46]. In addition, an in vitro experimental study showed that the combination of harringtonine (HT) and L-asp did not play a synergistic role in the treatment of AML [47]. A potential reason is that L-asp is easily inactivated by lysosomal cysteine protease B (CTSB) and asparagine endopeptidase produced in the bone marrow microenvironment [48]. Therefore, if the protective effect of bone marrow microenvironment could be overcome, the anti-AML activity of L-asp would achieve better outcome.

### Glutamine uptake inhibitors

It was demonstrated that the glutamine transporter was increased in most cancer tissues [28–30]. Considering the dependence of AML on glutamine, inhibition of glutamine transporter can be another strategy for AML treatment. So far, the most studied glutamine transporter is SLC1A5, a Na^+^ transmembrane transporter that mediates the transport of amino acids such as cellular glutamine [40, 49]. It was reported that SLC1A5, a high-affinity cell membrane transporter for glutamine, was highly expressed in various types of cancer cells including AML cell lines MOLM-14, MV4-11, OCI-AML3, and HL-60 [50]. Recently, studies have indicated that suppressing SLC1A5 by RNA interference or pharmacological inhibition can effectively prevent cancer development and progression in human AML xenograft mouse models, which appears to be associated with glutamine uptake and mTORC1 pathway [39, 50]. SLC1A5 promotes intracellular glutamine accumulation, which in turn increases leucine uptake via the SLC7A5 (LAT1) transporter, a process that is critical for activation of the mTORC1 signaling pathway via the Ragulator-Rag complex. Willems et al. found that glutamine deprivation inhibited mTORC1 activity and induced apoptosis in AML cells [39]. In a mouse model of leukemia, targeting SLC1A5 reduced the intracellular glutamine content and hindered the activation of mTORC1, which interfered with the energy supply, leading to the inhibition of cell growth and cell cycle progression [39]. Therefore, it is necessary to further investigate the combination of mTORC1 inhibitors with SLC1A5 inhibitors in AML treatment.

GPNA is a small molecule inhibitor that inhibits Na-ion-dependent amino acid transporters including SLC1A5. In xenograft models, GPNA can reduce the development and progression of leukemia driven by *MLL-AF9* or *PTEN* deficiency, suggesting that SLC1A5 is a promising therapeutic target for the treatment of leukemia [50]. V-9302 is a competitive antagonist of transmembrane glutamine flux and selectively targets SLC1A5. It is shown to have anticancer effects and is in preclinical trials at present. The combination of V-9302 and glutaminase inhibitor depletes glutathione and induces ROS production, leading to apoptosis in hepatocellular carcinoma cells [51]. Li et al. found that V-9302 inhibited the cell viability in a dose-dependent manner and promoted the apoptosis of AML cell lines HL-60 and KG-1 [52]. Together, to fully elucidate the role of transporter protein in AML pathogenesis provides potential drug targets for new therapies.

SLC38, the major transporter protein of glutamine, contributes to the maintenance of human homeostasis [31]. Previous studies have demonstrated that knockdown of SLC38A1 significantly reduces the proliferation and metastatic capacity of cancer cells, including AML cells [30, 32]. A recent study indicated that high SLC38A1 expression was an independent adverse prognostic biomarker in AML. Yan Li et al. examined SLC38A1 expression in 277 AML patients and found that patients with high SLC38A1 expression had a higher risk of adverse karyotypes and a shorter overall survival [30]. Gilteritinib is a tyrosine kinase inhibitor approved for the treatment of relapsed/refractory AML with FLT3 mutations. Recently, Zavorka’s group investigated the mechanism of gilteritinib in AML treatment. Treating AML mice with gilteritinib resulted in the decrease of SLC38A1 transcripts in a time-dependent manner. Additionally, knockdown of SLC38A1 by RNA interference led to a significant reduction in glutamine uptake. Thus, gilteritinib exhibits anti-AML effects probably through reducing SLC38A1 and subsequently inhibit glutamine metabolism [33]. These results reveal that a drug inhibiting glutamine transporter expression may be therapeutically beneficial in targeting cellular metabolism.

### Glutamine antagonists/analogues

Glutamine antagonists/analogues including 6-diazo-5-oxo-norleucine (DON), acivicin, and JHU-083, are substances that are highly similar to glutamine in structure and capable of selectively blocking multiple metabolic reactions in which glutamine is involved. They are considered to be drugs that can effectively target glutamine metabolism and show great anticancer effects [34, 53]. DON competitively binds to the glutamine active site, forming a covalent compound that irreversibly inhibits a variety of glutamine-metabolizing enzymes. Van Gastel et al. found that applying DON at short or continuous 1-day interval in AML mice after treatment with induction chemotherapy (iCT) method increased the rate of AML cell elimination. They also proved that treatment of mice with DON led to glutamine accumulation, indicating that DON was able to inhibit glutamine metabolism in AML cells [54]. These findings suggest that activation of glutamine metabolism plays a role in protecting AML cells after iCT treatment, and suggest the potential application value of controlling glutamine metabolism to continuously eliminate AML cells. In addition, DON can inhibit cellular nucleotide production and result in cytotoxicity against AML [55]. Rosefeld et al. demonstrated that DON induced HL-60 cell differentiation and inhibit their proliferation [53].

Acivcin, another glutamine antagonists/analogue, inhibits the activity of enzymes catalyzing glutamine reactions and depletes certain cellular metabolites. Studies have confirmed the anti-cancer effects of acivcin in LI210 [53] and P388 [56] leukemia mice. Moreover, acivcin has been shown to facilitate differentiation of myeloid leukemia cells, inhibit cancer cell proliferation, and induce cell apoptosis in a time- and dose-dependent manners [56]. However, these glutamine analogues caused serious neurotoxicity, gastrointestinal reactions, and other side effects [36, 57].

Recently, JHU-083, a small molecule designed on the basis of DON, has become an important glutamine analogue targeting glutamine metabolism with high oral availability and low toxicity for normal cells. It can cross the blood–brain barrier, and prolong the survival of medulloblastoma mice. Furthermore, JHU-083 has been reported to improve the bone marrow microenvironment and promote immune cells to restore their immune responses [34]. Although studies on aspects related to JHU-083 in AML have not yet been conducted, we believe that this inhibitor will be beneficial to AML patients.

### Glutaminase inhibitors

Over the past decades, many strategies targeting glutamine metabolism have been proposed in AML treatment. Inhibition of glutaminase activity has become a major focus of academic and pharmacological cancer metabolism research [58, 59]. Gutaminase (GLS) is a mitochondrial enzyme catabolizing glutamine to glutamate, which is the first catalytic step in cellular glutamine metabolism. The *GLS* gene encodes two isozymes, “kidney-shaped” (GLS1) and “liver-shaped” (GLS2) [9]. GLS2 is considered to be a tumor suppressor while GLS1 has the potential to facilitate tumor development [58, 60]. In recent years, investigators have found that the GLS1 isoform GAC is highly expressed in different types of cancers such as breast cancer, pancreatic cancer, and malignant leukemia [18]. In AML, GLS is overexpressed in primary cells. The expression of GLS1 is significantly higher than that of GLS2 from the genomic profiling database [20, 61]. Inhibition of GLS was found to promote apoptosis and suppress the growth and proliferation of AML cells [61, 62]. The importance of GLS in AML cells is probably due to the following reasons: GLS catalyzes the intracellular glutamine to glutamate that is further catabolized to α-KG. This process is essential for the TCA cycle and oxidative phosphorylation in AML cells [11, 19, 58]. Gregory et al. found that either glutamine depletion or inhibition of GLS activity decreased the rate of ATP and oxygen consumption and ultimately induced apoptosis in AML cells [11]. When AML primary cells, cell lines, and normal CD34 hematopoietic cells were cultured in the medium with or without glutamine, lack of glutamine inhibited the proliferation and survival of AML cells but had no significant inhibitory effects on CD34 hematopoietic cells. The inhibitory effects on AML cells could be partially or completely reversed by α-KG, confirming the relationship between glutaminolysis and TCA cycle [11]. Moreover, this study also indicated that GLS inhibition significantly impaired the production of antioxidant glutathione in types of AML cell lines, leading to increased mitochondrial ROS (mitoROS) and cell apoptosis [11, 17]. Overall, these results demonstrate that targeting glutaminases can be a potential therapeutic option for the treatment of AML.

GLS is highly expressed in various malignancies and has been widely studied as a target for cancer therapy. Several GLS inhibitors such as compound 968, BPTES, and CB-839 are currently in preclinical studies [19, 61]. Compound 968 is a small molecule agent, which is a metamorphic modulator of GLS1 and is able to inhibit the activity of GLS1 splice variants, KGA, and GAC [63]. More and more studies have investigated the effects of compound 968 on the cancer treatment in vivo and in vitro. For example, compound 968 was reported to block various Rho-GTPase-induced carcinogenesis in fibroblasts without toxic effects on normal cells [35]. Yuan L found that compound 968 inhibited glutamine metabolism by suppressing the activity of GLS and thus greatly inhibited the proliferation of three breast cancer cell lines [64]. In addition, a number of experiments in vitro have revealed that compound 968 has anticancer activity against endometrial cancer, ovarian cancer, and small cell lung cancer (SCLC) [37, 64]. Therefore, compound 968 may provide new therapeutic strategies for the treatment of different types of cancers. However, it remains unclear whether compound 968 plays a similar role in AML cells.

Bis-2-(5-phenylacetamido-1, 2, 4-thiadiazol-2-yl) ethyl sulfide (BPTES) is a reversible inhibitor of metastable glutaminase and inhibits the activation of GLS1. Since BPTES lacks the structural similarity to glutamine, it has fewer off-target effects caused by interaction with glutamine-related enzymes [38]. The anti-leukemic activity of BPTES has been proved in vitro. Sontakke P et al. found that BPTES at concentrations of 20 μM and 40 μM not only reduced the oxygen consumption and oxidative phosphorylation capacity, but also suppressed TCA cycle by inhibiting cellular glutamine metabolism, leading to the inhibition of cell growth and proliferation in leukemic cell lines with BCR- ABL mutations [65]. In another study, BPTES was able to inhibit the growth of AML cells at concentrations of 10 μM and 20 μM [5]. Furthermore, it was reported that BPTES inhibited the growth of AML primary cells with IDH mutations [18]. Therefore, BPTES has exhibited great anti-leukemic activity.

CB-839, an oral small molecule inhibitor of GLS, is developed on the basis of BPTES and being evaluated as a drug for solid tumors and hematologic malignancies [66]. IDH is an enzyme to convert α-KG into the oncogenic metabolite 2-HG. AML cells with the IDH1/2 mutations are highly sensitive to the inhibition of enzymes in glutamine metabolism. CB-839 can reduce the 2-HG in AML cells with the IDH1/2 mutation and induced myeloid differentiation [61]. Additionally, glutamine metabolism supports the mitochondrial functions and cellular redox metabolic capacity of AML cells, resulting in the adverse prognosis of AML patients carrying the FLT3-ITD mutation. Notably, CB-839 also showed inhibitory effects on AML cells with FLT3 mutation [67]. Zacharias et al. found that ATP levels were significantly decreased in HL-60 and OCI-AML3 cell lines in the presence of 1 μM CB-839 for 72 h. Interestingly, CB-839 inhibited the proliferation of OCI-AML3 cells rather than HL-60 cells [68]. Currently, CB-839 has been in phase I and phase II clinical trials on AML (ClinicalTrials.gov: NCT02071927, ClinicalTrials.gov: NCT03047993).

However, GLS inhibitors have limited activity when used as monotherapy, and subsequent studies have focused on combination therapies. It is proved that GLS inhibitors can exert synergistic anti-leukemia effects with adjuvant drugs that disturb mitochondrial redox status, such as arsenic trioxide (ATO) and homeopathic erythromycin (HHT). Indeed, the combination of CB-839 with ATO or HHT exacerbates mitochondrial oxidation and apoptosis in the cell lines, mouse models, and patients of AML [11]. In addition, GLS inhibitors are effective in killing AML cells when acting synergistically with other chemotherapeutic agents. In mouse models of FLT3 mutant AML, a combination treatment of CB-839 and AC220 (FLT3 inhibitor) prolonged survival by approximately 1 week in comparison to that of treatment with AC220 alone. The mechanism may be that CB-839 and AC220 synergistically deplete glutathione, induce the production of mitochondrial ROS, and lead to apoptosis. Besides, Gregory et al. found that AC220 was able to modulate the glycolytic function but showed few inhibitory effects on TCA cycle in AML cells [19]. However, combination of CB-839 and AC220 can inhibit the TCA cycle and restricted cell growth greatly [19, 67]. Moreover, the use of CB-839 in combination with ABT-199 (BCL-2 inhibitor) facilitated mitochondrial depolarization, decreased cellular oxidative phosphorylation capacity, and induced apoptosis in OCI-AML2 and MORM-14 cell lines [20]. Additionally, cytosine arabinoside (Ara-c) combined with CB-839 or BPTES inhibited the proliferation of leukemic cells more strongly than that of monotherapy [5, 17]. Thus, it is necessary to conduct clinical researches to explore new chemotherapeutic combinations of GLS inhibitors in the treatment of AML.

## Discussion

Preclinical studies have proved the anti-leukemic effects of targeting glutamine metabolism. However, the inhibitors of glutamine metabolism should be used cautiously in AML treatment due to their limitations at the time of clinical trials.

Targeting glutamine metabolism reduces mitochondrial oxidative phosphorylation capacity and inhibits the TCA cycle in AML cells. However, exogenous α-KG can restore mitochondrial oxidative phosphorylation capacity, and alternative supply pathways of TCA cycle can be upregulated, both leading to resistance of glutamine-related inhibitors. Moreover, although targeting glutamine metabolism inhibits the entry of carbon catabolized by glutamine into the TCA cycle, it was found that the glycolytic process is not altered by targeting glutamine metabolism in AML cells, implying that the products required for the TCA cycle are not affected [16]. In the last few years, studies have shown that fatty acid oxidation (FAO) has provided an additional supply pathway for the TCA cycle in leukemia stem cells (LSCs). One explanation may be that FAO produces acetyl coenzyme A and citrate through the mitochondria, and acetyl coenzyme A can produce NADPH through a series of enzymatic reactions [69, 70]. In addition, recent studies have demonstrated that AML cells can use lactic acid as a carbon source in homeostasis to provide raw materials for the TCA cycle of AML cells to maintain cell growth and proliferation [71]. Therefore, future research targeting glutamine metabolism should focus on how to inhibit multiple metabolic pathways without affecting normal cells to achieve effective treatment for AML.

Moreover, studies have identified multiple glutamine metabolic pathways in AML cells. Each part of the glutamine metabolic pathways can be regulated by multiple factors. Thus, interference with a single glutamine metabolic pathway or one part of that may have minimal effects in treating AML. For instance, Polet et al. found that SLC38A2 was significantly upregulated as a compensatory transporter of glutamine when SLC1A5 was absent or inhibited in leukemia cells [28]. Besides, inhibition of SLC1A5 expression did not induce apoptosis in HL-60 cells, possibly as a result of the presence of other intracellular glutamine transporters [39]. Nemkov et al. revealed that direct reduction of glutamine exerted a stronger anti-leukemic effect compared to inhibition of GLS activity in FLT3-ITD mutant cells, suggesting that FLT3-ITD mutant cells may rely on a paracrine pathway of glutamine metabolism for glutamate production [19]. In addition, GLS2 is an often overlooked role in tumor glutamine metabolism. The function of GLS2 is regulated both by various oncogenes and tumor suppressor genes. N-MYC, an important member of the MYC family, can directly accelerate the activation of GLS2 rather than GLS1 and promote oxidative glutamine metabolism in *MYCN*-amplified neuroblastoma cells. Depletion of GLS2 decreased the α-KG, ATP, and GSH and suppressed the cell proliferation and viability in vitro and in vivo [72]. Interestingly, it was shown that the tumor suppressor p53 also induced GLS2 expression in response to DNA damage or oxidative stress and thus promoted the glutamine metabolism and decrease ROS levels. Overexpression of GLS2 inhibited tumor cell growth and colony formation, implicating GLS2 as a contributor to p53-mediated tumor suppression [73]. Furthermore, GLS2 that is upregulated by GATA3 in luminal-subtype breast cancer modulated the resistance to GLS inhibitors such as BPTES. However, another GLS inhibitor, the small molecule 968, can suppress GLS2 effectively and inhibit cell growth and proliferation in BPTES-resistant breast cancer [74]. These results indicate that GLS2 can act as a double-edged sword in regulating cellular activities, which depends on upstream signals.

The inhibitors of glutamine metabolism have complex effects on the immune responses in cancer patients. Actually, glutamine plays a key role both in cancer cells and immune cells. Similar to cancer cells, immune cells use glutamine metabolites to accelerate the cellular TCA cycle and oxidative phosphorylation processes, thus rapidly obtaining the energy for growth and immune action [54]. Additionally, studies in vitro and vivo demonstrated that glutamine significantly mediated the proliferation and cytokine production of lymphocytes, phagocytosis and secretory activity of macrophages, and the capacity of neutrophils [75]. Sornsuvit C et al. uncovered that parenteral supplementation of glutamine enhanced the phagocytosis of neutrophils in AML patients [76]. Inhibition of glutamine metabolism can lead to the immune escape of cancer cells. Previous studies showed that BPTES promoted the upregulation of PD-L1 expression, which can inhibit the anticancer effects of immune cells. Interestingly, BPTES also accelerated the production of pro-inflammatory cytokines produced by M1-like macrophages and boosted the immune responses [77, 78]. In human breast cancer cell lines, the glutamine transporter inhibitor V-9302 upregulated PD-1 expression and induced immune escape [78]. It is a promising strategy to overcome drug resistance and improve the efficacy of chemotherapy in AML patients through targeting glutamine metabolism. However, this strategy may disrupt the anticancer effects of immune cells in the bone marrow microenvironment. Therefore, how to target glutamine metabolism in cancer cells without disturbing immune responses will be a great challenge in the subsequent studies.

In conclusion, the available studies suggest that targeting glutamine metabolism can inhibit mitochondrial oxidative phosphorylation, reduce glutathione production, disrupt redox homeostasis, and inhibit mTORC1 activation in AML cells. The attractive therapeutic strategies include the use of glutamine depletion, glutamine uptake inhibitors, glutamine antagonists/analogues, and GLS inhibitors. In the subsequent study, we should systematically investigate and further characterize glutamine metabolism in AML, and develop efficient drugs targeting glutamine metabolism without side effects for AML treatment.

## References

[1] Acute myeloid leukaemia (2016). Nat Rev Dis Primers.

[2] Newell LF, Cook RJ (2021). Advances in acute myeloid leukemia. BMJ.

[3] Bose P, Vachhani P, Cortes JE (2017). Treatment of relapsed/refractory acute myeloid leukemia. Curr Treat Options Oncol.

[4] LeBlanc TW, Erba HP (2019). Shifting paradigms in the treatment of older adults with AML. Semin Hematol.

[5] Gronningsaeter IS, Reikvam H, Aasebo E, Bartaula-Brevik S, Tvedt TH, Bruserud O, et al. Targeting cellular metabolism in acute myeloid leukemia and the role of patient heterogeneity. Cells. 2020;9(5). 10.3390/cells9051155. 10.3390/cells9051155 PMC7290417 32392896

[6] Altman BJ, Stine ZE, Dang CV (2016). From Krebs to clinic: glutamine metabolism to cancer therapy. Nat Rev Cancer.

[7] Mayers JR, Vander Heiden MG (2015). Famine versus feast: understanding the metabolism of tumors in vivo. Trends Biochem Sci.

[8] Yang L, Venneti S, Nagrath D (2017). Glutaminolysis: a hallmark of cancer metabolism. Annu Rev Biomed Eng.

[9] Darmaun D, Matthews DE, Bier DM (1986). Glutamine and glutamate kinetics in humans. Am J Physiol.

[10] Rex MR, Williams R, Birsoy K, Ta Llman MS, Stahl M (2022). Targeting mitochondrial metabolism in acute myeloid leukemia. Leuk Lymphoma.

[11] Gregory MA, Nemkov T, Park HJ, Zaberezhnyy V, Gehrke S, Adane B (2019). Targeting glutamine metabolism and redox state for leukemia therapy. Clin Cancer Res.

[12] Kreitz J, Schonfeld C, Seibert M, Stolp V, Alshamleh I, Oellerich T, et al. Metabolic plasticity of acute myeloid leukemia. Cells. 2019;8(8). 10.3390/cells8080805 10.3390/cells8080805 PMC6721808 31370337

[13] Meng D, Yang Q, Wang H, Melick CH, Navlani R, Frank AR (2020). Glutamine and asparagine activate mTORC1 independently of Rag GTPases. J Biol Chem.

[14] Jones CL, Stevens BM, D’Alessandro A, Reisz JA, Culp-Hill R, Nemkov T (2018). Inhibition of amino acid metabolism selectively targets human leukemia stem cells. Cancer Cell.

[15] Wang D, Tan G, Wang H, Chen P, Hao J, Wang Y (2019). Identification of novel serum biomarker for the detection of acute myeloid leukemia based on liquid chromatography-mass spectrometry. J Pharm Biomed Anal.

[16] Saito Y, Sawa D, Kinoshita M, Yamada A, Kamimura S, Suekane A (2020). EVI1 triggers metabolic reprogramming associated with leukemogenesis and increases sensitivity to L-asparaginase. Haematologica.

[17] Dernie F (2021). Characterisation of a mitochondrial glutamine transporter provides a new opportunity for targeting glutamine metabolism in acute myeloid leukaemia. Blood Cells Mol Dis.

[18] Emadi A, Jun SA, Tsukamoto T, Fathi AT, Minden MD, Dang CV (2014). Inhibition of glutaminase selectively suppresses the growth of primary acute myeloid leukemia cells with IDH mutations. Exp Hematol.

[19] Gregory MA, Nemkov T, Reisz JA, Zaberezhnyy V, Hansen KC, D’Alessandro A (2018). Glutaminase inhibition improves FLT3 inhibitor therapy for acute myeloid leukemia. Exp Hematol.

[20] Jacque N, Ronchetti AM, Larrue C, Meunier G, Birsen R, Willems L (2015). Targeting glutaminolysis has antileukemic activity in acute myeloid leukemia and synergizes with BCL-2 inhibition. Blood.

[21] Yang WH, Qiu Y, Stamatatos O, Janowitz T, Lukey MJ (2021). Enhancing the efficacy of glutamine metabolism inhibitors in cancer therapy. Trends Cancer.

[22] Amaya ML, Inguva A, Pei S, Jones C, Krug A, Ye H (2022). The STAT3-MYC axis promotes survival of leukemia stem cells by regulating SLC1A5 and oxidative phosphorylation. Blood.

[23] Weng H, Huang F, Yu Z, Chen Z, Prince E, Kang Y (2022). The m(6)A reader IGF2BP2 regulates glutamine metabolism and represents a therapeutic target in acute myeloid leukemia. Cancer Cell.

[24] Zhao H, Jiang Y, Lin F, Zhong M, Tan J, Zhou Y (2022). Chidamide and apatinib are therapeutically synergistic in acute myeloid leukemia stem and progenitor cells. Exp Hematol Oncol.

[25] Emadi A, Law JY, Strovel ET, Lapidus RG, Jeng LJB, Lee M (2018). Asparaginase Erwinia chrysanthemi effectively depletes plasma glutamine in adult patients with relapsed/refractory acute myeloid leukemia. Cancer Chemother Pharmacol.

[26] Emadi A, Kapadia B, Bollino D, Bhandary B, Baer MR, Niyongere S (2021). Venetoclax and pegcrisantaspase for complex karyotype acute myeloid leukemia. Leukemia.

[27] Michelozzi IM, Granata V, De Ponti G, Alberti G, Tomasoni C, Antolini L (2019). Acute myeloid leukaemia niche regulates response to L-asparaginase. Br J Haematol.

[28] Polet F, Martherus R, Corbet C, Pinto A, Feron O (2016). Inhibition of glucose metabolism prevents glycosylation of the glutamine transporter ASCT2 and promotes compensatory LAT1 upregulation in leukemia cells. Oncotarget.

[29] Rosilio C, Nebout M, Imbert V, Griessinger E, Neffati Z, Benadiba J (2015). L-type amino-acid transporter 1 (LAT1): a therapeutic target supporting growth and survival of T-cell lymphoblastic lymphoma/T-cell acute lymphoblastic leukemia. Leukemia.

[30] Li Y, Shao H, Da Z, Pan J, Fu B (2019). High expression of SLC38A1 predicts poor prognosis in patients with de novo acute myeloid leukemia. J Cell Physiol.

[31] Broer A, Rahimi F, Broer S (2016). Deletion of amino acid transporter ASCT2 (SLC1A5) reveals an essential role for transporters SNAT1 (SLC38A1) and SNAT2 (SLC38A2) to sustain glutaminolysis in cancer cells. J Biol Chem.

[32] Wang K, Cao F, Fang W, Hu Y, Chen Y, Ding H (2013). Activation of SNAT1/SLC38A1 in human breast cancer: correlation with p-Akt overexpression. BMC Cancer.

[33] Zavorka Thomas ME, Lu X, Talebi Z, Jeon JY, Buelow DR, Gibson AA (2021). Gilteritinib inhibits glutamine uptake and utilization in FLT3-ITD-positive AML. Mol Cancer Ther.

[34] Hanaford AR, Alt J, Rais R, Wang SZ, Kaur H, Thorek DLJ (2019). Orally bioavailable glutamine antagonist prodrug JHU-083 penetrates mouse brain and suppresses the growth of MYC-driven medulloblastoma. Transl Oncol.

[35] Wang JB, Erickson JW, Fuji R, Ramachandran S, Gao P, Dinavahi R (2010). Targeting mitochondrial glutaminase activity inhibits oncogenic transformation. Cancer Cell.

[36] Lemberg KM, Vornov JJ, Rais R, Slusher BS (2018). We’re Not “DON” yet: optimal dosing and prodrug delivery of 6-Diazo-5-oxo-L-norleucine. Mol Cancer Ther.

[37] Guo H, Li W, Pan G, Wang C, Li D, Liu N (2023). The glutaminase inhibitor compound 968 exhibits potent in vitro and in vivo anti-tumor effects in endometrial cancer. Anticancer Agents Med Chem.

[38] Robinson MM, McBryant SJ, Tsukamoto T, Rojas C, Ferraris DV, Hamilton SK (2007). Novel mechanism of inhibition of rat kidney-type glutaminase by bis-2-(5-phenylacetamido-1,2,4-thiadiazol-2-yl)ethyl sulfide (BPTES). Biochem J.

[39] Willems L, Jacque N, Jacquel A, Neveux N, Maciel TT, Lambert M (2013). Inhibiting glutamine uptake represents an attractive new strategy for treating acute myeloid leukemia. Blood.

[40] Zhang Z, Liu R, Shuai Y, Huang Y, Jin R, Wang X (2020). ASCT2 (SLC1A5)-dependent glutamine uptake is involved in the progression of head and neck squamous cell carcinoma. Br J Cancer.

[41] Chan WK, Horvath TD, Tan L, Link T, Harutyunyan KG, Pontikos MA (2019). Glutaminase activity of L-Asparaginase contributes to durable preclinical activity against acute lymphoblastic leukemia. Mol Cancer Ther.

[42] Chen T, Zhang J, Zeng H, Zhang Y, Zhang Y, Zhou X (2020). Antiproliferative effects of L-asparaginase in acute myeloid leukemia. Exp Ther Med.

[43] Buaboonnam J, Cao X, Pauley JL, Pui CH, Ribeiro RC, Rubnitz JE (2013). Sequential administration of methotrexate and asparaginase in relapsed or refractory pediatric acute myeloid leukemia. Pediatr Blood Cancer.

[44] Wells RJ, Woods WG, Lampkin BC, Nesbit ME, Lee JW, Buckley JD (1993). Impact of high-dose cytarabine and asparaginase intensification on childhood acute myeloid leukemia: a report from the Childrens Cancer Group. J Clin Oncol.

[45] Ohnuma T, Holland JF, Nagel G, Arneault GS (1969). Effects of L-asparaginase in acute myelocytic leukemia. JAMA.

[46] Onuma T, Waligunda J, Holland JF (1971). Amino acid requirements in vitro of human leukemic cells. Cancer Res.

[47] Okano T, Ohnuma T, Holland JF, Koeffler HP, Jui H (1983). Effects of harringtonine in combination with acivicin, adriamycin, L-asparaginase, cytosine arabinoside, dexamethasone, fluorouracil or methotrexate on human acute myelogenous leukemia cell line KG-1. Invest New Drugs.

[48] Kaspers GJL (2019). Acute myeloid leukaemia niche regulates response to L-asparaginase. Br J Haematol.

[49] Liu Y, Zhao T, Li Z, Wang L, Yuan S, Sun L (2018). The role of ASCT2 in cancer: a review. Eur J Pharmacol.

[50] Ni F, Yu WM, Li Z, Graham DK, Jin L, Kang S (2019). Critical role of ASCT2-mediated amino acid metabolism in promoting leukaemia development and progression. Nat Metab.

[51] Jin H, Wang S, Zaal EA, Wang C, Wu H, Bosma A, et al. A powerful drug combination strategy targeting glutamine addiction for the treatment of human liver cancer. Elife. 2020;9. 10.7554/eLife.56749. 10.7554/eLife.56749 PMC7535927 33016874

[52] Li QQ, Pan SY, Chen QY, Zhou W, Wang SQ. [Effect of competitive antagonist of transmembrane glutamine flux V-9302 on apoptosis of acute myeloid leukemia cell lines HL-60 and KG-1]. Zhongguo Shi Yan Xue Ye Xue Za Zhi. 2021;29(3):685–9. 10.19746/j.cnki.issn.1009-2137.2021.03.005. 10.19746/j.cnki.issn.1009-2137.2021.03.005 34105457

[53] Rosenfeld H, Roberts J (1981). Enhancement of antitumor activity of glutamine antagonists 6-diazo-5-oxo-L-norleucine and acivicin in cell culture by glutaminase-asparaginase. Cancer Res.

[54] van Gastel N, Spinelli JB, Sharda A, Schajnovitz A, Baryawno N, Rhee C (2020). Induction of a timed metabolic collapse to overcome cancer chemoresistance. Cell Metab.

[55] Lyons SD, Sant ME, Christopherson RI (1990). Cytotoxic mechanisms of glutamine antagonists in mouse L1210 leukemia. J Biol Chem.

[56] Ardalan B, Arakawa M, Villacorte D, Jayaram H, Cooney DA (1982). Effect of L-glutamine antagonists on 5-phosphoribosyl 1-pyrophosphate levels in P388 leukemia and in murine colon adenocarcinomas in vivo. Biochem Pharmacol.

[57] Earhart RH, Amato DJ, Chang AY, Borden EC, Shiraki M, Dowd ME (1990). Phase II trial of 6-diazo-5-oxo-L-norleucine versus aclacinomycin-A in advanced sarcomas and mesotheliomas. Invest New Drugs.

[58] Masisi BK, El Ansari R, Alfarsi L, Rakha EA, Green AR, Craze ML (2020). The role of glutaminase in cancer. Histopathology.

[59] Wang S, Yan Y, Xu WJ, Gong SG, Zhong XJ, An QY (2022). The role of glutamine and glutaminase in pulmonary hypertension. Front Cardiovasc Med..

[60] Liu J, Zhang C, Lin M, Zhu W, Liang Y, Hong X (2014). Glutaminase 2 negatively regulates the PI3K/AKT signaling and shows tumor suppression activity in human hepatocellular carcinoma. Oncotarget.

[61] Matre P, Velez J, Jacamo R, Qi Y, Su X, Cai T (2016). Inhibiting glutaminase in acute myeloid leukemia: metabolic dependency of selected AML subtypes. Oncotarget.

[62] Jacque N, Bouscary D (2014). Targeting glutamine uptake in AML. Oncoscience..

[63] Yu W, Yang X, Zhang Q, Sun L, Yuan S, Xin Y (2021). Targeting GLS1 to cancer therapy through glutamine metabolism. Clin Transl Oncol.

[64] Yuan L, Sheng X, Clark LH, Zhang L, Guo H, Jones HM (2016). Glutaminase inhibitor compound 968 inhibits cell proliferation and sensitizes paclitaxel in ovarian cancer. Am J Transl Res.

[65] Sontakke P, Koczula KM, Jaques J, Wierenga AT, Brouwers-Vos AZ, Pruis M (2016). Hypoxia-like signatures induced by BCR-ABL potentially alter the glutamine uptake for maintaining oxidative phosphorylation. PLoS ONE.

[66] Gross MI, Demo SD, Dennison JB, Chen L, Chernov-Rogan T, Goyal B (2014). Antitumor activity of the glutaminase inhibitor CB-839 in triple-negative breast cancer. Mol Cancer Ther.

[67] Gallipoli P, Giotopoulos G, Tzelepis K, Costa ASH, Vohra S, Medina-Perez P (2018). Glutaminolysis is a metabolic dependency in FLT3(ITD) acute myeloid leukemia unmasked by FLT3 tyrosine kinase inhibition. Blood.

[68] Zacharias NM, Baran N, Shanmugavelandy SS, Lee J, Lujan JV, Dutta P (2019). Assessing metabolic intervention with a glutaminase inhibitor in real-time by hyperpolarized magnetic resonance in acute myeloid leukemia. Mol Cancer Ther.

[69] Carracedo A, Cantley LC, Pandolfi PP (2013). Cancer metabolism: fatty acid oxidation in the limelight. Nat Rev Cancer.

[70] Samudio I, Harmancey R, Fiegl M, Kantarjian H, Konopleva M, Korchin B (2010). Pharmacologic inhibition of fatty acid oxidation sensitizes human leukemia cells to apoptosis induction. J Clin Invest.

[71] Erdem A, Marin S, Pereira-Martins DA, Geugien M, Cunningham A, Pruis MG (2022). Inhibition of the succinyl dehydrogenase complex in acute myeloid leukemia leads to a lactate-fuelled respiratory metabolic vulnerability. Nat Commun.

[72] Xiao D, Ren P, Su H, Yue M, Xiu R, Hu Y (2015). Myc promotes glutaminolysis in human neuroblastoma through direct activation of glutaminase 2. Oncotarget.

[73] Suzuki S, Tanaka T, Poyurovsky MV, Nagano H, Mayama T, Ohkubo S (2010). Phosphate-activated glutaminase (GLS2), a p53-inducible regulator of glutamine metabolism and reactive oxygen species. Proc Natl Acad Sci U S A.

[74] Lukey MJ, Cluntun AA, Katt WP, Lin MJ, Druso JE, Ramachandran S (2019). Liver-type glutaminase GLS2 is a druggable metabolic node in luminal-subtype breast cancer. Cell Rep.

[75] Ma G, Zhang Z, Li P, Zhang Z, Zeng M, Liang Z (2022). Reprogramming of glutamine metabolism and its impact on immune response in the tumor microenvironment. Cell Commun Signal.

[76] Sornsuvit C, Komindr S, Chuncharunee S, Wanikiat P, Archararit N, Santanirand P (2008). Pilot study: effects of parenteral glutamine dipeptide supplementation on neutrophil functions and prevention of chemotherapy-induced side-effects in acute myeloid leukaemia patients. J Int Med Res.

[77] Nabe S, Yamada T, Suzuki J, Toriyama K, Yasuoka T, Kuwahara M (2018). Reinforce the antitumor activity of CD8(+) T cells via glutamine restriction. Cancer Sci.

[78] Liu PS, Wang H, Li X, Chao T, Teav T, Christen S (2017). Alpha-ketoglutarate orchestrates macrophage activation through metabolic and epigenetic reprogramming. Nat Immunol.

